# Facial Reconstruction Using a Bilobed Flap Following Extensive Resection of Right Hemifacial Squamous Cell Carcinoma

**DOI:** 10.7759/cureus.89126

**Published:** 2025-07-31

**Authors:** Irvint Joel Bautista Perez, Uriel Alejandro Guillen Morales, Enrique Perez Robles

**Affiliations:** 1 General Surgery, Universidad Nacional Autonoma de Mexico, Mexico City, MEX; 2 General Surgery, Hospital General "Dr. Fernando Quiroz Gutiérrez" Instituto de Seguridad y Servicios Sociales de los Trabajadores del Estado (ISSSTE), Mexico City, MEX; 3 Plastic Surgery, Hospital General "Dr. Fernando Quiroz Gutiérrez" Instituto de Seguridad y Servicios Sociales de los Trabajadores del Estado (ISSSTE), Mexico City, MEX

**Keywords:** bilobed flap, facial reconstruction, reconstruction technique, squamous cell carcinoma, tumour

## Abstract

The facial region has always posed a challenge in terms of the type of flap that could be used to cover facial defects secondary to multiple etiologies. Over time, the search has been on for a highly versatile flap, either because it can be harvested from multiple sites or because it provides security in terms of irrigation. One example is the bilobed flap for nasal reconstruction, which can be used over time with great versatility in different sites on the face. This flap is characterized by two lobes that share a single vascular pedicle, allowing for rotational coverage of defects with minimal risk of necrosis. While its traditional use was primarily for nasal defects, recent modifications and clinical adaptations have expanded its application to atypical facial locations such as the cheek, temporal region, and mandible.

This report presents a clinical case of an 85-year-old male patient with a long-standing, non-painful hyperpigmented lesion on the right cheek, which was diagnosed as a moderately differentiated squamous cell carcinoma. Due to the lesion's size and involvement of multiple facial subunits (zygomatic, temporal, and maxillary regions), a wide local excision was performed in conjunction with intraoperative pathology. Reconstruction was achieved using a bilobed flap sourced from the cheek. The surgical plan was designed to ensure the preservation of both function and facial aesthetics, critical considerations in geriatric oncology patients.

The discussion highlights the anatomical and vascular considerations essential for flap viability, including the rich vascular supply of the cheek from the transverse facial artery, facial artery perforators, and infraorbital branches. It also addresses the importance of preserving the facial nerve during flap elevation. The bilobed flap’s advantages, such as minimal donor site morbidity, predictable vascular supply, and good cosmetic outcomes, make it particularly suitable for elderly patients with comorbidities who may not tolerate more extensive reconstructions.

In conclusion, the bilobed flap remains a reliable and adaptable reconstructive technique for facial defects, including those resulting from oncologic resections. This case reinforces its utility in non-traditional locations and underlines the importance of individualized surgical planning. With proper anatomical understanding and technique, the bilobed flap offers an effective solution for restoring both form and function in complex facial reconstructions.

## Introduction

The original design of the bilobed flap was described by Esser in 1918 for nasal reconstruction. This flap consists of two lobes that share a single vascular pedicle. Subsequently, McGregor and Soutar were the first to report the potential variability in the angle between both lobes, initially reaching up to 90°. However, this angle introduced hinge deformity at the rotational point. In 1989, Zitelli introduced a modification using a 45° angle, not exceeding 90-100°, which resulted in improved outcomes. Among its variants, the typical movement involves a 45° rotation for the first lobe and a 90° rotation for the second, providing a total arc of 135°. The second lobe covers the defect left by the first; however, the forward advancement of the first lobe facilitates closure, and the defect of the second can be closed directly, especially if narrow [1].

Over time, the bilobed flap has been adapted to cover defects in regions other than the nose, such as frontal bilobed flaps (for large defects without brow or eyelid distortion), temporal bilobed flaps (for large defects without eyelid traction), mandibular bilobed flaps, and phalangeal applications. Literature highlights its versatility for large defect coverage, which led to its use in this case [2].

## Case presentation

An 85-year-old male patient, with chronic degenerative comorbidities including well-controlled type 2 diabetes mellitus and systemic arterial hypertension under control, reported the onset of a hyperpigmented lesion on the right cheek. It initially presented as a painless macule measuring approximately 0.5 x 0.5 cm, which was left to evolve freely over four years, eventually reaching dimensions of 15 x 12 cm, involving the zygomatic, temporal, and maxillary regions. He was referred to surgical oncology, where a punch biopsy was performed. The histopathological report indicated infiltrating, non-keratinizing, moderately differentiated squamous cell carcinoma, with no angio- or neuroinvasion identified, no necrosis, and depth not assessable. As a result, joint management with oncological surgery was initiated, including wide tumor excision with intraoperative histological analysis and reconstruction using a bilobed flap covering the maxillary region and extending to the neck, 2 cm below the mandibular ramus.

Procedure description

**Figure 1:**
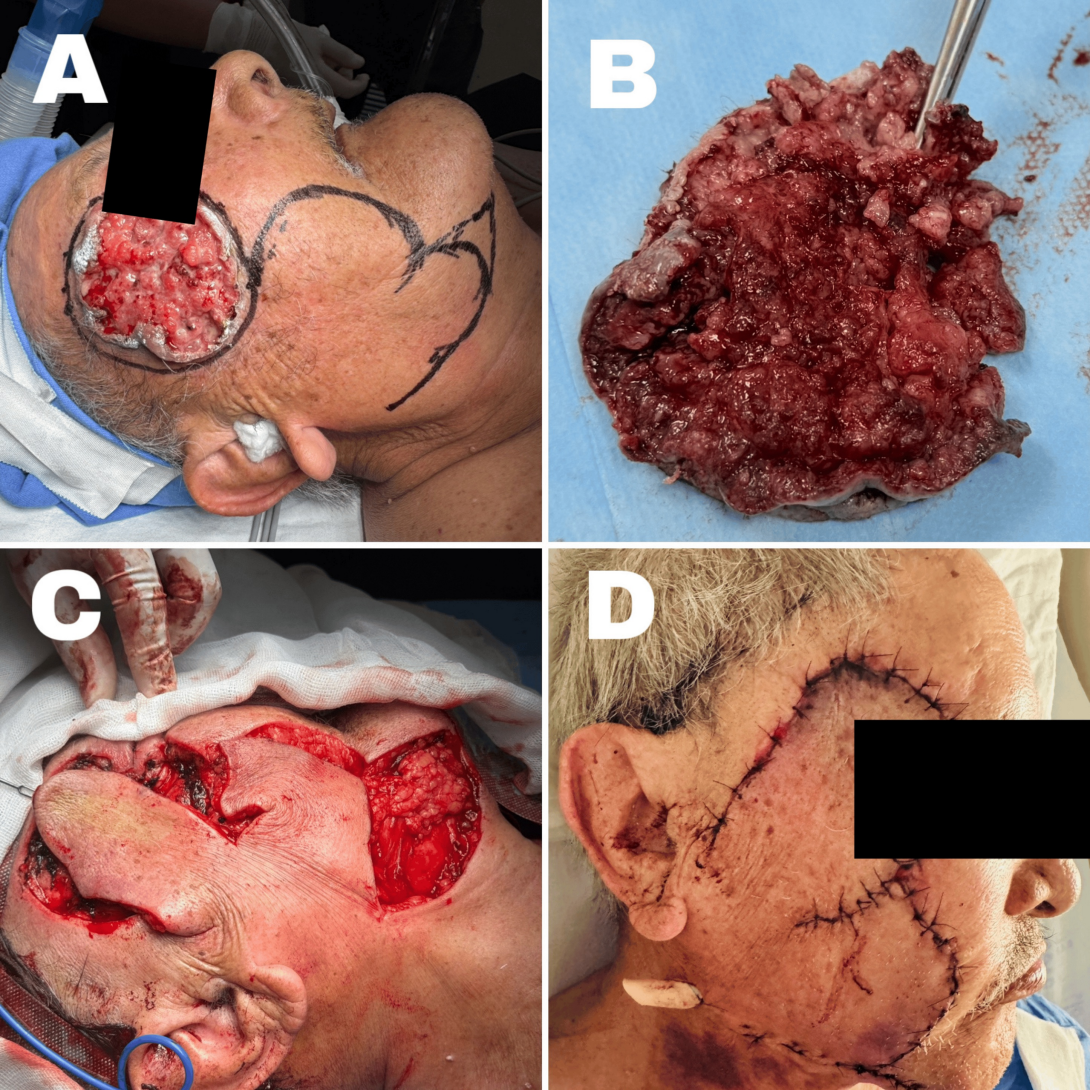
Reconstruction technique A: flap marking; B: squamous cell carcinoma; C: flap rotation; D: postoperative result Written informed consent to include this image in an open-access article was obtained from the patient.

Preoperative marking was performed (Figure 1A), delineating the tumor as well as the two lobes of the flap: the first lobe, located inferior to the lesion, was intended to cover the post-excisional defect; the second lobe would close the donor area from the first, and the lower margin was planned for direct closure. Wide resection of the skin lesion was carried out, yielding a circular tumor measuring 6.5 x 6 cm with a thickness of 1.5 cm (Figure 1B). It was sent for intraoperative pathological analysis, which reported clear margins, 0.5 cm laterally and 0.7 to 10 mm in depth, diagnosing ulcerated squamous cell carcinoma with focal superficial keratinization, moderately differentiated, <10% necrosis, and tumor-free margins.

**Figure 2:**
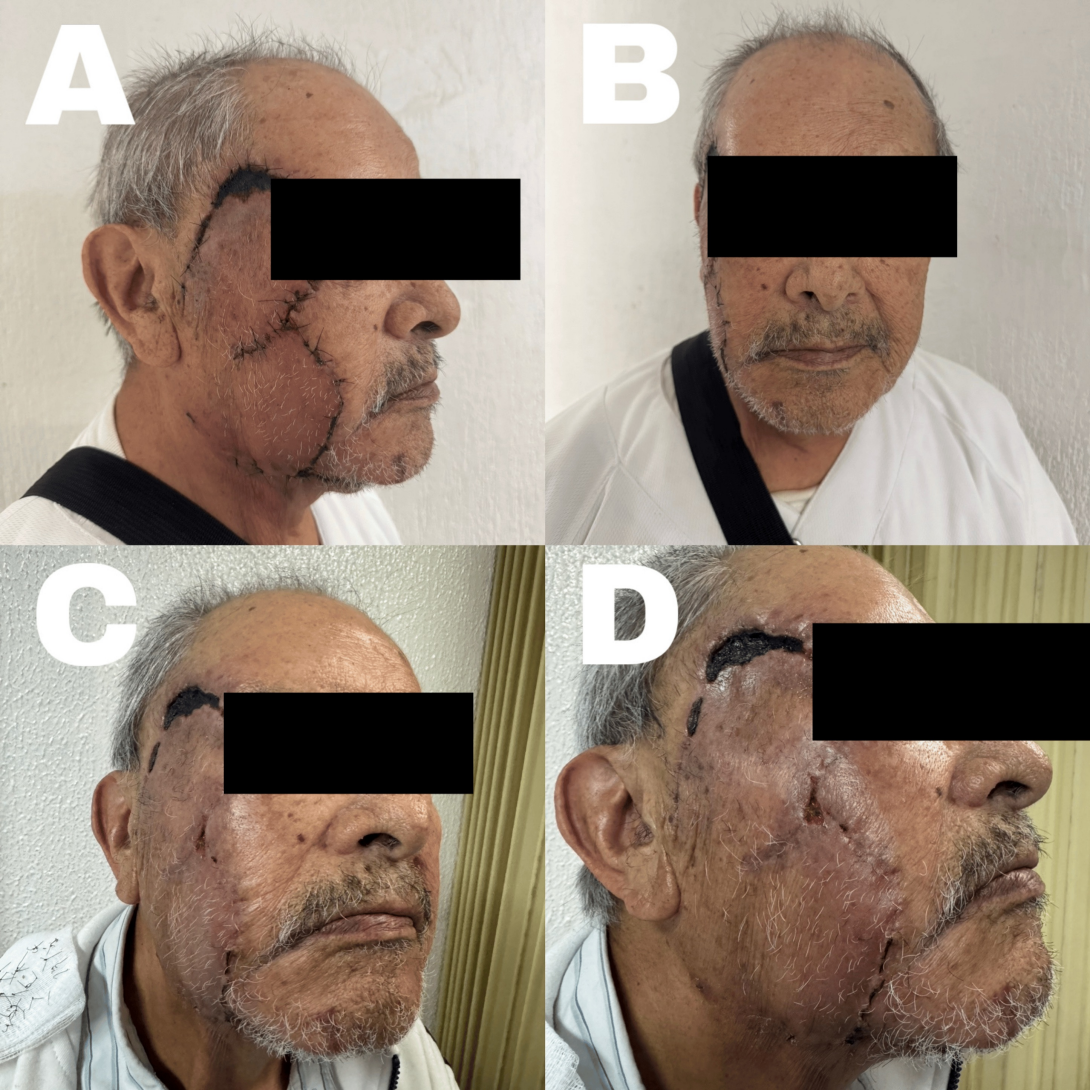
Postoperative follow-up A: 15 days postoperative, lateral view; B: 15 days postoperative, frontal view; C: one-month postoperative, with 90% flap integration; D: one-month postoperative, with the upper zone still in the process of integration Written informed consent to include this image in an open-access article was obtained from the patient.

A local partial-thickness subcutaneous flap was elevated (Figure 1C), rotated into place, and its coverage confirmed. Hemostasis was ensured, and closure was performed in layers using 2-0 Vicryl for subcutaneous sutures. A Y-shaped Penrose drain was inserted: one branch directed towards the subcutaneous surgical bed and the other towards the lower portion of the second lobe. Skin closure was completed using simple interrupted 3-0 nylon sutures. The patient showed an adequate immediate postoperative closure (Figure 1D). Follow-up at 15 days showed slight erythema of the flap (Figures 2A, 2B), and after one month, the bilobed flap showed 90% integration (Figures 2C, 2D).

## Discussion

Facial reconstruction procedures are widely employed in patients with sequelae from basal cell carcinomas, squamous cell carcinomas, melanomas, large benign tumors, or soft tissue trauma. The objective is to restore function and facial aesthetics, a key component of social interaction. Hence, planning with local flaps must consider facial aesthetic units, subunits, skin texture, and color [3].

Numerous facial flaps have been described, supported by the region’s rich vascular network. Rotation, island, advancement, and transposition flaps are the foundation of most facial reconstructions. Common donor-based flaps include the frontal flap, cheek rotation-advancement flap, cervicofacial flap, and nasolabial flaps. Flap selection depends on the affected aesthetic unit, and careful planning should incorporate natural lines, folds, and margins. Facial zones include the forehead, eyelids, cheeks, nose, lips, and chin [4].

Particular attention must be given to the facial nerve during flap planning. It exits the stylomastoid foramen and courses through the parotid gland, dividing it into superficial and deep lobes. The nerve splits into the temporo-zygomatic and cervico-facial branches. The former gives rise to buccal, zygomatic, temporal, and frontal branches; the latter, to mandibular and cervical branches. The flap’s vascular supply must also be considered for predictability, relying on axial vessels such as the facial, superficial temporal, supratrochlear, supraorbital, infraorbital, and labial arteries [5].

Facial tissues (epidermis, dermis, subcutaneous fat, and ligaments) vary in proportion and thickness across facial units and influence flap design. The cheek’s laxity makes it an ideal donor site for large flaps, providing robust coverage with low necrosis risk due to blood supply from the transverse facial artery, facial artery perforators, and minor infraorbital artery contributions. The transverse facial artery, a branch of the superficial temporal artery, courses through the parotid gland and across the masseter fascia to form a dense subdermal plexus with extensive arborisation and anastomoses with nasal artery branches [6].

In our case, the extensive defect involved the zygomatic and temporal regions. While a cervicofacial advancement flap was considered, patient age and comorbidities made this a salvage option should the bilobed flap fail. The cheek’s vascularity and literature-supported viability favored the bilobed flap. Only one similar case was found in the literature involving a bilobed flap for a squamous cell carcinoma [7]. Our procedure was successful, and the patient remains disease-free under oncological and medical follow-up. A short margin near the lateral canthus was left due to intraoperative considerations and the patient’s overall condition, avoiding a more morbid cervicofacial flap. The tumor was staged as T3N0M0, and the patient retains excellent facial aesthetics and social reintegration [8].

Squamous cell carcinoma is the second most common non-melanoma skin cancer, with 75-90% occurring in the head and neck. It is also the most metastasis-prone of these tumors. Treatment consists of wide excision, with or without cervical lymphadenectomy. Literature suggests that mortality correlates more with age than staging due to lymph node metastasis risk, and aggressiveness of treatment should consider individual factors rather than staging alone [9].

## Conclusions

Regardless of etiology, trauma, dermal lesion, or tumor, facial defects present a challenge to plastic and reconstructive surgery, as the face is central to social interaction. While tumor excision is essential, one must not overlook the psychological and social impact of facial disfigurement. Reconstruction planning should aim to restore appearance and integration into daily life.

Based on the literature, the bilobed flap is highly versatile, suitable for both small and large defects, and offers reliable outcomes due to the vascular richness of the face. Our patient continues to do well postoperatively, with excellent flap integration and no evidence of tumor recurrence.
